# Raising Adolescent Children as a Developmental Task of Iranian Middle-aged Mothers: A Qualitative Study

**DOI:** 10.30476/IJCBNM.2021.90710.1726

**Published:** 2022-04

**Authors:** Fatemeh Fallahi, Monireh Anoosheh, Mahshid Foroughan, Zohreh Vanaki, Anoshirvan Kazemnejad

**Affiliations:** 1 Department of Nursing, School of Medical Sciences, Tarbiat Modares University, Tehran, Iran; 2 Research Center on Aging, Department of Gerontology, University of Social Welfare and Rehabilitation Sciences, Tehran, Iran; 3 Department of Biostatistics, School of Medicine, Tarbiat Modares University, Tehran, Iran

**Keywords:** Adolescent, Middle-aged, Mothers, Parenting, Qualitative research

## Abstract

**Background:**

Most parents consider adolescence to be the most difficult stage of parenting. Parental practice is a determining factor in adolescents’ outcomes. Mothers play the main role of parenting in Iran. Coinciding the transition of adolescence with the transition of middle-aged mothers can affect the mothers’ parenting practice. The present study aimed at explaining the Iranian mothers’ practice in parenting an adolescent child.

**Methods:**

This qualitative conventional content analysis was conducted from July 2018 to November 2019 in Kashan. 21 in-depth semi-structured interviews with mothers of adolescent children were performed using a purposive sampling method. The data were analyzed through conventional content analysis. Data collection and analysis were performed simultaneously using MAXQDAv10 software.

**Results:**

Regarding the study objectives, two themes and six main categories were identified. The theme of ‘laying the groundwork for upbringing’ was detected by two main categories: ‘meeting the needs and ‘effective interaction with the adolescent’; also, the theme of ‘individual-social capacity building’ was explained by four main categories: ‘helping to gain independence’, ‘modeling individual-social behavior’, ‘socializing the adolescent’, and ‘preparing to accept future roles’.

**Conclusion:**

Mothers’ practice was mainly focused on adolescents’ independence, college education, career prospects, and marriage preparation and respect for older adults. Consistent with this transition to modernity, and contrary to the collectivist values of Iranian society, mothers’ practice was in line with developing adolescents’ independence and building their self-confidence, which is close to the authoritative parenting style.

## INTRODUCTION

Parenting practice has been proven to be closely related to many aspects of adolescent development. Poor parental supervision, lack of support, and inefficient parent-adolescent relationship lead to psychosocial incompatibility of adolescents. ^1^ Most parents consider their child’s adolescence period to be the most difficult stage of parenting. ^2^ Adolescence is the stage of transition from childhood to adulthood and moving towards achieving identity and independence, new social roles, and responsibility in various areas of life. This transition can be challenging for adolescents and their parents. ^3^ Usually, the child’s adolescence coincides with the parents’ middle age. ^4^ The world has seen rapidly growing middle-aged population over the past decade. ^5^ In Iran, this age transition, which is accompanied by an increase in the middle-aged population, is taking place as well. ^6^ Parenthood in middle age often involves managing biological, cognitive, emotional, and psychosocial changes. This period is often accompanied by increasing responsibility at home and work. Adolescent puberty may pose additional developmental challenges to the current situation of middle-aged parents, especially mothers. ^4^

Middle-aged mothers experience more challenges than fathers. Biological changes associated with middle age (menopause, changes in health status, and changes due to aging) and social and psychological challenges make this period more critical for women. ^7^ A study in the United States (2018) showed that middle-aged mothers considered the problems related to adolescent parenting and coping with family changes as the most important challenges of this period. Balancing work and personal life, changing family relationships, managing multiple family responsibilities, and rediscovering oneself were other important challenges for middle-aged women. At the same time, they have to take care of their spouses, teenage children, and elderly family members, so they are called the “sandwich generation”. ^8^ Mothers face the developmental challenges of adolescence along with their middle-aged developmental changes. Coinciding the transition of adolescence with that of middle-aged mothers can affect the mothers’ parenting practice. ^9^

Contrary to the popular belief that adolescents are aliens to their parents, it has been shown that they have a deep relationship and strong attachment to their parents, especially their mothers. ^10^ Mothers have a higher level of support, more responsiveness, and more behavioral control over their adolescents. ^11^ In a study, adolescents have stated that their mothers know them better, provide more guidance, and are more involved in their issues than their fathers. Particularly in eastern societies such as Iran, where historically authoritarian parenting style is more common, children feel closer to their mothers. In Iran, women play a key role in managing the home and raising children. ^10^

Iran has moved towards modernity in recent years. ^12^ The emergence of modernity which was accompanied by changes in socialization patterns, earlier onset of puberty, prolonged education, and delay in employment and marriage has led to prolongation of adolescence. Age definitions are always arbitrary, and chronological approaches to the definition of adolescence will continue to be shaped by culture and context. Despite clear definitions of adolescence, newer views suggest that current definition of adolescence is overly restricted because of the widely spread postponement of role transitions to adulthood, and prolongation of adolescence. Nowadays, an expanded definition and timeframe of adolescence (often up to about 24 years of age) aligns more closely and is better fitted with contemporary patterns of adolescent development. On the one hand, lengthening adolescence and delay in adulthood has caused today’s adolescents to experience a greater degree of transition and crisis than before. Moreover, lengthening adolescence means that adolescents rely on the parents and families for longer periods. Consequently, parental practice is a key factor for adolescents’ successful transition to adulthood. ^13^

Also, modernization has made the task of raising an adolescent child more difficult due to rapid socio-cultural changes and the effects of multiple information sources on adolescents. ^14^ Simultaneously, the development of urbanization and industrialization, higher education, employment, and economic capital of Iranian women have led to a reconsideration of the role of motherhood and the value of the child. Iranian women prefer to be better mothers for their fewer children. ^15^

However, despite modernity, there is still a traditional adherence to the limited definition of gender roles in Iranian society. The general opinion is that raising a child is the responsibility of mothers. Besides, in Iran, families are deeply influenced by religious teachings. ^12^ Motherhood is one of the most important roles of a woman, according to Islamic culture; to properly adjust to the motherly role, family roles take precedence over social roles, and the main developmental task of them as mothers is to raise good children. ^16^ Developmental tasks are defined as age-graded normative tasks based on societal expectations that should be reached in a specific life phase. In this regard, one of the most important developmental tasks for middle-aged mothers is to help the adolescent child to become a happy and responsible adult. ^17^ Successful attainment of this developmental task leads to psychological well-being of middle-aged mothers. ^9^

Surprisingly, research focusing on middle-aged mothers’ parenting practice has been rather limited. Few studies conducted in the field of middle-aged parenting show that the quality of communication between adolescents and parents is the main predictor of the parents’ sense of competence. The focus of these studies was on the mental health, quality of life, and well-being of middle-aged parents, not their parenting practice. ^4 , 9^ The studies in the field of parenting have mainly used a quantitative approach, focusing on parenting challenges, parenting styles, and the effects of parenting on adolescent outcomes including academic achievement, delinquency and behavioral disorders, and self-confidence. ^1 , 18 , 19^ In a qualitative study which aimed at explaining the parents’ perceptions of raising adolescent, parents stated that parent-child conflicts and difficulty in controlling adolescents are their concerns in performing the task of parenting during this developmental period. ^12^ However, what is less addressed in these studies is what strategies middle-aged mothers use to carry out their parenting task? Also, due to the increase in the middle-aged mother population in Iran, rapid modernization, more developmental challenges, and given the information gap in this area, researchers conducted this qualitative study to explain the Iranian mothers’ practice in parenting the adolescent child. 

## MATERIALS AND METHODS

This study was conducted based on the naturalistic research paradigm, with a qualitative design and conventional content analysis approach. The content analysis focuses on the life experience, interpretations, and meanings that individuals have encountered. ^20^ The current study was conducted on 18 mothers and lasted for 16 months from July 2018 to November 2019. The present study was conducted in Kashan in the central part of Iran. Middle-aged women (40-59 years old) with at least one adolescent child (10 to 19 years old) ^21^ participated in the study. The inclusion criteria were married middle aged women with healthy adolescent child, no mental disorders, willingness to participate in the study and express their experiences, and the ability to understand and speak in the Persian language. The only exclusion criterion was the mother’s unwillingness to continue the study at any time during the research. Sampling was done by purposive method until data saturation was reached. Data were collected using semi-structured individual face-to-face interviews. 

The study setting was 9 urban health centers of Kashan, Iran. In these health centers, in the framework of the health system reform plan, a middle-aged women’s health service package is designed, and electronic health records are created for these women. To receive routine care, they periodically refer to these centers. Health center staff are aware of these women’s social characteristics and have access to their telephone numbers and health records. The researcher had access to the participants with the guidance of the managers and staff of these centers. 

The first meeting with each participant served to introduce the study, explain the purpose of the study, inquire if they were interested in participating, and schedule a suitable time and place for the following session. The interviews were performed in the mothers’ preferred places, such as schools, homes, parks, or health centers. All interviews were conducted in the Persian Language and by the first author, who was trained in conducting qualitative interviews. Each interview began with a general question, *“Could you tell me about your experiences with raising your teen?”*, *“Explain what you do to raise your adolescent,”*; based on the type of answers, the interviews were followed by other questions. To get deeper information, in-depth questions, such as *“What do you mean?* *Explain more! Can you give an example?”* were also used. Each interview lasted on average 55 minutes. A small portion of the two interviews (fifth, and fifteenth participants) was clarified with three ten-minute telephone conversations. All interviews (21 interviews with 18 participants) were recorded with the consent of the participants, and at the earliest opportunity, they, along with nonverbal cues, gestures, and moods, were typed word-by-word in Microsoft Word.

Two field notes were used as a method of data collection. The researcher was sensitive to events in various environments in society, and especially in health centers; in case incidental observations and events related to the subject were made, the researcher recorded them in the form of a field note. These events and observations, immediately after the exposure, were typed and analyzed. 

After typing the text, all interviews were entered into Maxqda software version 10 to facilitate data organization. The analysis was performed simultaneously with data collection, using the conventional content analysis approach. The text of each interview was read and reviewed several times. To analyze the data, we adopted Zhang and Wildermuth’s method (2017) due to its transparent and systematic procedure. ^22^ To this end, after preparing the text, meaning units were identified according to the research question, and appropriate codes were written for each meaning unit. After three interviews, the primary codes were categorized and named based on conceptual similarity (sub-categories), and after being reviewed, modified, and confirmed by the research team, the process of data collection and analysis continued. The sub-categories were compared and placed in more abstract main categories (categories). The main categories were then conceptualized, and a more abstract concept was assigned to them (theme); finally, the methodology and findings were reported. All the extracted codes and categories were reviewed and approved by the second and third authors.

To ensure the rigor of the findings, we used Lincoln and Guba’s (1985) four criteria. ^23^ Credibility was established through prolonged engagement with the data (more than a year was devoted to data collection and analysis) and member checking. In member checking, five interview transcripts together with their related primary codes were provided to the participants to check if our generated codes accurately reflected their experiences. To verify the confirmability of the findings, the interview texts, codes, and extracted categories were peer-checked and approved by the first and second authors and an outsider faculty member. To ensure the dependability of the findings, we recorded the steps and research processes and reported as accurately and step-by-step as possible. In this study, efforts were made to have a variety of participants in terms of age, job, education, and number of children, which in addition to credibility, helped to confirm the transferability of the findings.

### Ethical Considerations

This study was approved by the Ethics Committee of Tarbiat Modares University, Tehran, Iran (approval cod: IR.MODARES.REC.1397.027). Informed consent for participation in the study was obtained from the participants after explaining the purpose and the method of the study. Participants were also assured of the confidentiality of the information and audio files, and it was stated that they could withdraw from the study at any stage with no harm. No participant expressed reluctance to cooperate before or during the interview. The location and time of the interview were decided based on the participants’ opinions. The convenience and privacy of the interviewees were taken into account during the interview. A code was also assigned to each interview to protect the participants’ identities.

## RESULTS

The majority of participants were in the age group of 40 to 50 years old, employed and had a bachelor’s degree. Many of them had two adolescent children of both sexes. The demographic characteristics of the participants are shown in Table 1.

**Table 1:** Demographic characteristics of the participants (N=18)

No	Age	Marital status	Job	Education	Number of children	Child Sex
1	53	Married	Farmer	Diploma	3	Both sex
2	42	Married	Housewife	Elementary	2	Female
3	43	Married	Midwife	Bachelor	2	Both Sex
4	53	Married	Retired	Bachelor	2	Male
5	44	Married	Housewife	Elementary	3	Both sex
6	42	Married	Housewife	Elementary	3	Both sex
7	51	Married	Retired	Bachelor	4	Female
8	50	Married	Housewife	Elementary	3	Both sex
9	40	Married	Librarian	Master	2	Female
10	54	Married	Faculty member	Ddoctorate	2	Female
11	47	Married	Midwife	Bachelor	1	Male
12	48	Married	Part-time teacher	Ddoctorate	2	Both Sex
13	45	Married	Faculty member	Ddoctorate	2	Male
14	45	Married	Teacher	Bachelor	2	Male
15	48	Married	Housewife	Diploma	2	Both Sex
16	46	Married	Housewife	Bachelor	1	Female
17	40	Married	Housewife	Bachelor	2	Both sex
18	41	Married	Tailor	Diploma	3	Male

After conducting 21 interviews with 18 participants, 980 primary codes, 24 subcategories, and 6 main categories were extracted, out of which the 2 themes of ‘laying the groundwork for upbringing’ and ‘individual-social capacity building’ were extracted (Table 2).

**Table 2:** Extracted categories and subcategories

Subcategory	Main category	Theme
Meeting the adolescent’s emotional needs	Meeting needs	Laying the groundwork for the upbringing
Maintaining the adolescent’s health		
Meeting the adolescent’s financial needs		
Establishing a close relationship with the adolescent	Effective interaction with the adolescent	
Having calm and logical behavior toward the adolescent		
Respecting the adolescent’s privacy		
Building trust in the adolescent		
Teaching empathy with parents to the adolescent		
Supervising the adolescent’s social activities indirectly		
Developing independence in the adolescent	Helping to gain independence	Individual-social capacity building
Assigning responsibility to the adolescent		
Boosting the adolescent’s confidence		
Modeling individual behavior	Modeling individual-social behavior	
Modeling social-citizenship behavior		
Educating social - citizenship order	Socializing the adolescent	
Educating order in personal affairs		
Informing the adolescent about social norms		
Training to help the needy people		
Educating how to interact with others		
Creating opportunities to interact with society		
Preparing the adolescent for marriage	Preparing to accept future roles	
Educating the adolescent about financial and economic affairs		
Motivating the adolescent to study		
Boosting the adolescent’s ability to judge properly		

### 1) Laying the Groundwork for the Upbringing

According to the participants’ statements, the theme *“laying the groundwork for upbringing”*, demonstrates the provision of the necessary conditions and situations to help the adolescents achieve maximum capacity and flourish their latent talents. In this regard, one of the mothers stated: *“I could never talk to my own mother. She treated me so badly that I could not ask any questions at all. I never wanted this to happen to my children in a way that they could not be intimate with me. The groundwork should be prepared in such a way that the child talks about both his sorrows and his joys.”* Thus, the theme *laying the groundwork for upbringing* consisted of two categories: ‘meeting needs’ and ‘effective interaction with the adolescent’. 

#### 1.1. Meeting Needs

Meeting the adolescent’s needs included meeting emotional and financial needs and efforts to maintain the adolescent’s health. Mothers stated that they used verbal and nonverbal expressions of affection to meet the adolescent’s emotional needs.

*“We express our love mostly by kissing or, for example, warm greetings.” (P1)*. They also considered maintaining the adolescent’s physical and mental health. *“I am careful about my sons’ weight. I signed both of them up for a swimming training class. I’m sensitive about their food and nutrition. Or, for example, when I see my son is anxious during exam days, I talk to him and calm him down, and when he’s worried, I pay more attention to him” (P4)*. Moreover, mothers indirectly met their adolescents’ financial needs by providing facilities and financial support. *“As far as we can, we provide facilities for them. I will do my best to provide the things they want and need.” (P3)*.

### 1. 2. Effective Interaction with the Adolescent

The category of effective interaction with adolescents included establishing a heartfelt intimate relationship, as well as having a calm and logical attitude toward adolescents, respecting adolescents’ privacy, supervising their social relations and activities indirectly, giving conditional freedom to adolescents, educating them to empathize with their parents, and building trust in adolescents. 

The majority of the mothers stated that they were trying to create a friendly atmosphere, in which the adolescent could be comfortable and share his/her problems with them. *“We are very friendly. My children can easily speak their heart out with me. We don’t do anything in secrecy, or don’t fear each other.” (P2)*. In difficult situations, for coping with the adolescent, mothers used the techniques of interactive conversation, silence, and mediation between the father and the child. They respected their adolescent’s privacy by providing solutions such as not imposing their opinions on them and keeping their secrets. *“Some moms, in front of the teacher and the other kids, say: hey, my kids are annoying me. They talk about everything in front of the kids. However, I manage to resolve the issues between ourselves at home. I try not to let our secrets leak.” (P9)*. Also, mothers tried to gain their adolescents’ trust by adapting their words and behavior and keeping their promises. 

Mothers tried to establish a constructive conversation with the adolescent. Depending on the situation, they used a variety of techniques such as a direct and clear expression of opinions. *“give my idea directly and try to express my views directly on the issues as much as possible.” (P14)*. Also, mothers try to convince him/her using age-appropriate reasoning techniques as well as attracting the father’s cooperation in managing the challenging situations. For example, *“I justify her with a reason, for example, if she wants something that we cannot do; I tell her we cannot do it because of this” (P3)*. 

Mothers stated that they used indirect monitoring strategies such as: setting sensible limitations, and restrictions on freedom. *“I am in touch with his friend, acquaintance and those around him. I monitor his friends. What kind of person are those my kid is associating with?” (P18)*. 

### 2. Individual-social Capacity Building

Based on the mothers’ statements, they tried to do their parenting developmental task by developing and strengthening adolescent’s individual-social capabilities. In this regard, one of the mothers said: *“I support them so that their talent flourishes. For example, one day my son said that he wanted to set up a coffee net with my friend. I did not resist at all. We had a little help to get them started. We didn’t get into it much anymore. We let him stand on his own feet.” (P14)*. Thus, the theme of the individual-social capacity building included four categories: helping to gain independence, modeling individual-social behavior, socializing, and preparing to accept future roles.

#### 2. 1. Helping to Gain Independence

Three categories of developing independence, assigning responsibility, and boosting adolescent’s self-confidence represented the experiences of mothers in helping adolescents to gain independence. The majority of mothers stated that they gave independence to the adolescents in proportion to their age and tried to respect the adolescent’s freedom of making decisions and avoid excessive support. *“At the age of puberty and adolescence, children want to be independent. This is an inherent matter that children have. It’s gradually forming inside them. We shouldn’t prevent it. I didn’t prevent it” (P13)*.

To boost adolescents’ self-confidence, mothers used strategies such as encouraging them, not comparing them with their peers, and informing adolescents about individual differences. *“I say to him that exam scores mustn’t be so important to him. Not all people are the same. One gains higher score on one occasion and the other has lower score, and it may turn to reverse next time” (P3)*.

Moreover, the mothers gave the adolescents responsibilities in proportion to their abilities. *“I ask them to make a meal or iron their clothes themselves. Sometimes, I send my son to do shopping.” (P5)*. It is worth noting that there were few participants who stated that they did all of their adolescent’s work and were unwilling to delegate any responsibilities to them.

### 2. 2. Modeling Individual-social Behavior

The behavior modeling category included modeling individual behavior, social-citizenship behavior. Mothers stated that they trained their adolescents by becoming role models for individual, social, and citizenship behaviors. *“Well, kids see everything, for example, what their mother does, or what their father does. Most of the time, there’s no need to say it. My mother-in-law underwent an operation. I was the only one who was there from the beginning to the end. My daughter sees that I dress her wound and clean her house every day.” (P12)*.

In addition, they tried to be a role model for the adolescent by showing good conduct and effective communication with others. *“I wear make-up, I dress well, I try to be very polite to everyone, although I’m not in a good mood. My children see me and consider me as a role model.” (P5)*.

### 2. 3. Socializing the Adolescent

Socializing the adolescent category included educating social - citizenship order, education of order in personal affairs, educating social norms, training to help the needy people, educating to interact well with others, and creating opportunities for interacting with the community.

Mothers advised the adolescents to protect the environment, observe environmental health, and care for the law and order in society. *“I told him that he couldn’t drive unless he got his driver’s license. I told him to respect the people on the street and, also, told him he must stay in line when he wants to buy bread. We’re not different from others. They must put the trash outside door on time.” (P12)*.

The mothers said they taught their adolescents self-discipline by emphasizing the importance of maintaining order at home and advising them on planning for meeting their daily needs. They encouraged the teenagers to follow the norms of society, and also advised them to help the needy people. *“I tell them that they have to help the needy. We must not enclose ourselves in a world of our own.” (P13)*.

Talking of older adults’ expectations from younger ones, they taught the adolescents to care for and respect the elderly. *“If their grandfather gets angry at them, I tell my children that this is related to his age and they shouldn’t talk back, at all. He’s old, and they should consider his age.” (P6)*.

By advising to be tolerant and treat people well, put oneself in others’ shoes, not to judge others, help others without expecting the same response, and not retaliate the others’ misbehavior, they taught the adolescents the right way to interact with people. They also created the proper conditions for adolescents to interact with their peers by providing enough opportunities for peer interactions. *“My daughter has cut her relationships with most of her friends. She says this one talks like this, or that one is talking like that. But I told her that she must talk to everyone based on her/his condition.” (P10)*.

### 2. 4. Preparing to Accept Future Roles

This category included preparing the adolescents for marriage, training on financial management, motivating them to study, and fostering the adolescents’ judgment ability. The mothers did prepare their adolescents for marriage by stating key criteria for choosing a life partner and guiding them on how to deal with their spouse in future. *“For example, sometimes I say my daughter how a wife must love her husband. Or I say to her be careful and get ready for your future.” (P7)*. It is essential to note that the majority of mothers taught their adolescents about the role and importance of cultural, religious, social, and economic similarity, as well as maintaining a positive interaction with the family of their spouse. *”I said to them about the marriage; You do not think marriage is a deal. Marry someone who is culturally similar to you.” (P15)*. In addition, through involving the adolescent in family economic situations and affairs and educating them how to keep the balance between the income and expenses, the mothers emphasized the importance of financial management. They also promoted the adolescent’s motivation to study by emphasizing the value of science and knowledge and prioritizing education. *“I always encourage them to study so that they can better understand the world and have a better job position in society, telling them, look at how useful some people are.” (P17)*.

## DISCUSSION

This study was conducted to explain the role of maternal parenting in raising adolescents in Iranian culture. The findings showed that the mothers make efforts to lay the groundwork for the upbringing and building the individual-social capacity of their adolescents. As to the theme of “Laying the groundwork for the upbringing” which has the dimensions of “meeting needs” and “effective interaction with adolescent”, previous studies have reported a set of findings; meeting the adolescents’ needs reflects the mothers’ responsibility to them. ^24^ The emotional need is one of the most important needs of children. Mothers strengthen he adolescents’ confidence and social growth by expressing love, respect, and instilling a sense of security in them. ^25^ A close look at the literature shows that adolescents have reported the need for emotional support as one of the basic needs for maintaining their health. ^24^ Effective interaction with adolescents is one of the parenting skills that has been emphasized in optimal parenting styles. Findings from several studies have shown that parents who use the authoritative parenting style with dual characteristics of responsiveness (discipline, supervision, control) and demandingness (acceptance, friendliness, support, and parental involvement) raise more successful adolescents. ^1 , 18^ On the other hand, authoritarian parents would like their children to obey them and approve and use punitive methods. Permissive parents do not have a clear boundary for desirable and undesirable behaviors and refrain from control. Even though authoritative parents impose strict restrictions, their approach is warm and nurturing. They prefer reasoning compared to compulsion. ^1^ In this study alike, the mothers used age-appropriate reasoning in interaction with their adolescents. Reasoning is a method of control in which parents clarify their expectations, respect the adolescent’s perspective, justify their authority, and reduce the adolescent’s aggressive behavior. ^1^ In a study, adolescents considered the need for freedom under family supervision to be essential to their health. Adolescents emphasized the necessity of moderation in parental behavior and the indiscernible monitoring of their actions. ^24^ Applying logical restrictions, not imposing opinions, and respecting privacy provide a good ground for boosting the adolescent’s self-confidence and independence. ^19^ In this study, the mothers tried not to let a contradiction emerge between their words and behaviors to gain the adolescent’s trust. Such contradiction poses serious problems to the child and leads to the loss of trust in their parents. Building trust in adolescents increases the likelihood of compliance with the mother and prevents the adolescent from taking refuge in peers who, due to lack of experience, are not good counselors for them. ^26^

While in most Asian collectivist countries, families act in an authoritarian manner. ^27^ In the current study, mothers’ behaviors, such as warmth and support, reasoning, and effective interaction with the adolescent, indirect supervision of adolescent’s social activities, expression of love, and meeting emotional needs reflect the authoritarian parenting style. It seems that developing social roles of Iranian mothers, increasing the number of working, educated mothers, and having more access to mass media all have had a positive impact on their nurturing practice. ^15^ Collectivism means that individuals prioritize collective cultural values. In eastern cultures, which are predominantly collectivist, there are patriarchal views and segregation of gender roles. Traditional roles in the structure provide more opportunities for mothers to participate in parenting. ^10^ In these cultures, there are conservative values of obedience, politeness, and respect for authority; oriental cultural values are generally associated with less emotional expression in the family. ^27^ A qualitative study conducted in Ethiopia with the aim of explaining the values and experiences of parents in the upbringing of adolescent, the results showed that the majority of parents in adolescent upbringing pay attention to career success, education, respect and communication, culture, and family. Most parents expressed a desire to raise obedient children who accept their parents’ values. However, in Western cultures, which are largely characterized by individualistic values, self-esteem, creativity, initiative and independence are encouraged. ^28^

As to the theme of “ Individual - social capacity building “ which has four dimensions of “Helping to gain independence”, “Modeling individual-social behavior”, “Socializing the adolescent”, and “Preparing to accept future roles”, previous studies have reported a set of findings. Mothers are considered an important source of nurture and support for child independence. ^29^ In a survey in the United States, the majority of parents used at least one strategy, such as providing more options for adolescents and involving their children in certain activities to help develop the adolescents’ independence. However, one-fourth of these parents identified themselves as the main obstacle to adolescents’ access to independence. ^30^ The type of parenting practice in supporting independence is in contrast to collectivist values such as self-sacrifice, loyalty, respectfulness, maintaining harmonious relationships, and prioritizing group interests. ^29^ Culturally, Iran, similar to other Middle Eastern countries, is a collectivist society. In such cultures, the authoritarian parenting style is more common. Although Iranian society has a collectivist culture, the practice of mothers in our study was in line with the development of adolescents’ independence. The reason for this contradiction might be the fact that Iranian society is on the path to modernity. ^10^ Modernization- the expansion of urbanization, literacy, and the use of mass media- has led the individuals to change their behavior according to changes in the social context, leading to a decline in some values and their replacement by new ones. As society becomes more modern, individuals tend to act more individualistically. ^31^

Researchers have emphasized the importance of parental practices as role models, especially in adolescence. In a descriptive study, the majority of adolescents followed at least one role model. ^32^ Another study showed family members were the most commonly reported role model for adolescents. Adolescents who have identified a role model have higher levels of identity formation. ^33^ As previously shown, parental behavior is a major factor of value transfer and is more effective than words. ^28^ In the present study, mothers prepared the adolescents to be good citizens, good parents, and ready to accept adulthood roles by modeling themselves.

Despite numerous resources influencing adolescents, parents have been identified as the most important factor in their children’s socialization; ^34^ thus, parents are required to develop basic social skills such as empathy, cooperation, ability to establish and maintain relationships with peers, and adults. ^35^ In adolescence, the relationship with peers is an opportunity to practice social interaction skills, and the adolescents are happier and have better emotional regulation if they could have positive interactions with their peers. The peers can act as role models or be a source of social support. ^36^ Fortunately, the mothers of the present study were aware of the importance of socializing their adolescents. They try to socialize their adolescent children with different techniques. 

A noteworthy point in the findings of this study was the mothers’ practice, emphasizing showing and teaching respect for adults, especially the elderly. Respect for the elderly is a cultural and moral commitment and is emphasized in Iranian Islamic culture. Furthermore, in collectivist cultures, familial and filial piety, and respect for older people are one of the most closely held cultural values. ^37^

 Adolescents need guidance in various areas of marriage, employment, and responsible behavior. Guiding parents, who set boundaries and regulate the adolescents’ behaviors, plays a positive role in the child’s development. ^38^ In the socio-cultural context of Iran, parents, even after adolescence, have a major role in monitoring and guiding their children in issues such as choosing a field of study, a career, and a life partner, and also in exposure to life crises. ^24^ Similarly, in our study, the mothers encouraged their adolescents to make academic progress by motivating them. Higher education is equivalent to more progress, more job opportunities, and higher income. ^39^ Similar to Chinese parents and based on the collectivist culture of Iran, there is a more positive attitude towards educated people, and higher education is inherently a value. ^27^ Therefore, one of the mothers’ strategies to raise successful children was to increase their motivation for formal education. In Iran, parents feel responsible for their children’s lives at all stages of life, even in their adulthood. Children live with their parents until they marry and even during their adulthood. ^10^ Considering the importance of starting a family in collectivist cultures, ^40^ therefore, the mothers in our study taught their children how to choose a partner and how to run their married life. 

Finally, according to the mothers, their practice was mainly focused on developing the adolescents’ independence, behavioral modeling, socializing with emphasis on relationships with peers, educating them to respect adults and the elderly, and preparing them to accept future roles, especially in the field of education, profession, and marriage. In this study, the mothers practice was based on laying a groundwork for the upbringing and building adolescents’ individual-social capacity. They considered it their duty to raise happy and responsible adults by giving the adolescent the maximum capacity and flourishing their talents.

The strengths of this study were conducting in-depth interviews which was one of the most prominent rigors of the present study and provided the opportunity to ask follow-up questions, explore additional information, justify previous responses, and make connections between several topics. It also provided a comfortable space where individuals felt more comfortable to talk. Despite the fact that many studies have been done in the field of parenting, in the current study, the strategies of raising adolescents have been studied in detail and from developmental perspective.

This study also had some limitations that need to be mentioned. Mothers’ statements alone cannot provide a complete picture of the mothers’ practice in adolescent upbringing. It is suggested that adolescents’ and fathers’ perceptions of parenting practice in raising children should be explained in designing future studies. Furthermore, studies need to be designed and implemented with a deductive approach and based on one of the existing theories of parenting. In this study, gender stereotypes and gender socialization were not considered. The findings of this study are based on the mothers’ statements, which can be influenced by various factors such as psychological conditions, cultural limitations, and social acceptance. 

## CONCLUSION

Middle-aged mothers try to do their developmental task of parenting using positive parenting strategies. Parenting practice of mothers was focused on adolescents’ independence, college education, career prospects, marriage preparation, and respect for older adults. In line with the transition from tradition to modernity, the mothers’ practice in parenting is changing to authoritative. Consistent with this transition and contrary to the collectivist values of Iranian society, mothers’ practice was in line with developing independence in adolescents and building their self-confidence. Healthcare professionals, including family nurses that are in direct contact with families and mothers, can help improve maternal literacy and adolescents’ health by developing positive parenting educational programs; they can use the parenting strategies of mothers participating in this study to develop and promote culture-based parenting programs. It is recommended that future studies should explain the gender-based parenting practice. Also, parenting practices in single-parent families and those with special adolescents should also be examined.
